# Multimodality Imaging Demonstrates Reduced Right-Ventricular Function Independent of Pulmonary Physiology in Moderately Preterm-Born Adults

**DOI:** 10.1016/j.jcmg.2020.03.016

**Published:** 2020-09

**Authors:** Afifah Mohamed, Pablo Lamata, Wilby Williamson, Maryam Alsharqi, Cheryl M.J. Tan, Holger Burchert, Odaro J. Huckstep, Katie Suriano, Jane M. Francis, Joana Leal Pelado, Cristiana Monteiro, Stefan Neubauer, Philip T. Levy, Paul Leeson, Adam J. Lewandowski

Preterm-born individuals have altered right-ventricular (RV) structure and function in young adulthood (1). To what extent the pulmonary circulation impacts these findings remains largely unknown. However, unlike RV changes that are apparent across gestational ages of prematurity, acute and chronic pulmonary complications are primarily isolated to more extreme cases below 28 weeks’ gestation (2). Given that more than 80% of preterm births are moderately preterm—between 32 and 36 weeks’ gestation—understanding the extent of RV changes in this subpopulation are of increased public health interest. Accordingly, we used a detailed multimodal assessment to determine whether reductions in RV function are out of proportion to changes in pulmonary physiology in moderately preterm-born young adults.

We studied 101 normotensive participants aged 18 to 40 years (3). Of these, 54 were born at term (39.5 ± 1.4 weeks at birth), and 47 were born preterm (32.8 ± 3.2 weeks at birth). Echocardiography and cardiac magnetic resonance (CMR) were performed to characterize RV morphology, RV function, pulmonary hemodynamics, and RV-pulmonary arterial vascular coupling, as previously described (1,4). Creation of a RV statistical atlas of CMR images was undertaken adapting previously published methods (5). The end-diastolic frames of RV short-axis cine stacks with manually contoured endocardial contours were retrieved and rebuilt into binary segmentation images. Smooth meshes were fitted to the RV blood-pool anatomy, achieving subvoxel accuracy. The RV anatomy of each subject was then described with a mesh, and principal component analysis was undertaken to identify key modes of shape variation. Spirometry lung function tests were performed to measure forced expiratory volume in 1 s (FEV_1_) and forced vital capacity (FVC).

Statistical analysis was performed using SPSS Version 23 (IBM, Armonk, New York). All data were normally distributed, and Student's *t-*tests were used to compare continuous variables between the preterm-born and term-born adults, with adjustment for sex when appropriate. Multivariable linear regressions were completed to assess differences between groups for RV measures adjusting for sex, height, age, FEV_1_, and FVC; p values < 0.05 were considered significant.

**Figure 1:**
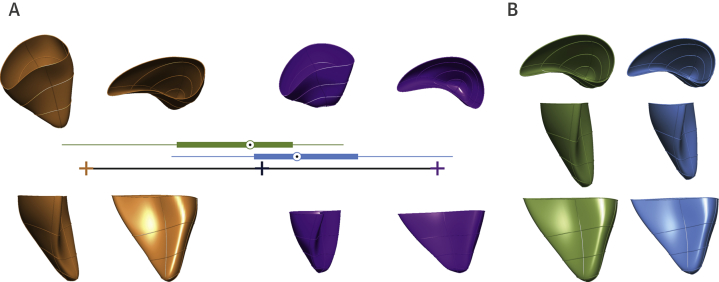
Unique RV Geometry **(A)** Principal component analysis coordinates for mode 1. The average across all individuals is set at coordinate 0 (**dark blue cross**). The **orange cross** and **orange** RV mesh represent –3SD, whereas the **purple cross** and **purple** RV mesh represent +3SD. Box plots represent the median mode 1 coordinates for each group (**blue** = preterm-born, and **green** = term-born). **(B**) Statistical average shape of the right ventricle of term-born **(green)** and preterm-born **(blue)** young adults.

RV end-diastolic areas and volumes were lower in preterm-born individuals (p ≤0.001). Measurements of RV function by echocardiography, including RV fractional area of change (FAC) and tricuspid annular plane systolic excursion (TAPSE), were lower in preterm-born compared with term-born adults (FAC: 38.91 ± 7.37% vs. 43.83 ± 7.01%; p = 0.008 and TAPSE: 1.84 ± 0.25 cm vs. 2.25 ± 0.35 cm; p < 0.001). Despite lower pulmonary artery acceleration times (PAATs) in those born preterm (141.1 ± 15.1 ms vs. 159.2 ± 21.6 ms; p < 0.001), indicating increased pulmonary vascular resistance, the RV remained coupled to its pulmonary circulation (TAPSE/PAAT: 0.13 ± 0.02 ms vs. 0.14 ± 0.03 m/s; p = 0.153). RV CMR revealed higher mass (21.20 ± 3.08 g/m^2^ vs. 18.98 ± 2.32 g/m^2^; p < 0.001) and lower ejection fraction (54.90 ± 5.17% vs. 57.48 ± 4.39%; p = 0.008) in those born preterm. Lower RV FAC, TAPSE, ejection fraction, and higher mass in preterm-born participants remained significant in multivariable regressions adjusting for pulmonary-function parameters (p < 0.05). Principal component analysis of the RV statistical atlas defined 5 anatomic modes of geometric variation within the study population, with mode 1 accounting for 25.3% of the variance. Preterm and term cohorts showed significant differences (p < 0.001) in mode 1, representing a smaller and shorter RV cavity in the preterm group, with no differences in other modes (Figure 1).

Although moderately preterm-born young adults exhibited structural and functional RV alterations, the RV remained coupled to the pulmonary vasculature. We speculate that uncoupling will be more likely to occur sooner in preterm-born individuals and may be gestational-age dependent. Our findings are of immediate public health concern and should be taken into clinical consideration, including regular, long-term follow-up of individuals born preterm. Future longitudinal research is needed to better understand individual patterns of cardiac remodeling throughout adulthood. Whether perinatal or later life clinical interventions known to improve RV physiology can modify the dysfunctional trajectory remains to be determined.
